# Women’s excess unhealthy life years: disentangling the unhealthy life years gap

**DOI:** 10.1093/eurpub/ckz114

**Published:** 2019-07-05

**Authors:** Wilma J Nusselder, Emmanuelle M Cambois, Dagmar Wapperom, France Meslé, Caspar W N Looman, Renata T C Yokota, Herman Van Oyen, Carrol Jagger, Jean Marie Robine

**Affiliations:** 1 Department of Public Health, Erasmus University Medical Center, Rotterdam, the Netherlands; 2 INED (French Institute for Demographic Studies), Mortality, Health, Epidemiology Research Unit, Paris, France; 3 SD Epidemiology and Public Health, Sciensano, Brussels, Belgium; 4 Department of Sociology, Interface Demography, Vrije Universiteit Brussel, Brussels, Belgium; 5 Department of Public Health and Primary Care, Ghent University, Ghent, Belgium; 6 Instiute of Health and Society, Newcastle University, Newcastle upon Tyne, UK; 7 Newcastle University Institute for Ageing, Newcastle upon Tyne, UK; 8 Inserm (French Institute of Health and Medical Research), CERMES3 Research Unit, Paris, France; 9 EPHE (École Pratique des Hautes Études), MMDN Research Unit, Univ., Montpellier, France

## Abstract

**Background:**

Compared to men, women live longer but have more years with disability. We assessed the contribution of gender differences in mortality and disability, total and by cause, to women’s excess unhealthy life years (ULYs).

**Methods:**

We used mortality data for France 2008 from Eurostat, causes of death from the CépiDc-INSERM-database; and disability and chronic conditions data from the French Disability Health Survey 2008–09. ULYs were calculated by the Sullivan method. The contributions of mortality and disability differences to gender differences in ULY were based on decomposition analyses.

**Results:**

Life expectancy of French women aged 50 was 36.3 years of which 19.0 were ULYs; life expectancy of men was 30.4 years of which 14.2 were ULYs. Of the 4.8 excess ULYs in women, 4.0 years were due to lower mortality. Of these 4.0 ULYs, 1.8 ULY originated from women’s lower mortality from cancer, 0.8 ULY from heart disease and 0.3 ULY from accidents. The remaining 0.8 excess ULY in women were from higher disability prevalence, including higher disability from musculoskeletal diseases (+1.8 ULY) and anxiety-depression (+0.6 ULY) partly offset by lower disability from heart diseases (−0.8 ULY) and accidents (−0.3 ULY).

**Conclusion:**

Lower mortality and higher disability prevalence contributed to women’s longer life expectancy with disability. Women’s higher disability prevalence due to non-fatal disabling conditions was partly offset by lower disability from heart disease and accidents. Conditions differentially impact gender differences in ULY, depending on whether they are mainly life-threatening or disabling. The conclusions confirm the health-survival paradox.

## Introduction

Women experience lower mortality, but report more disability compared to men of the same age. The female longevity advantage and the ‘health-survival paradox’ have received much attention in the scientific literature, but the fact that this implies that women live many more years with disability has received scant attention.^1–4^ In general, the focus of past research has been on total life expectancy and on healthy life expectancy, in Europe measured with the healthy life years (HLYs).^5^ Gender differences in HLY are generally much smaller than in life expectancy owing to opposite gender differences in mortality and disability which reduce the gap in HLY. In some countries, women have fewer HLYs than men indicating that their mortality advantage is offset by their disability disadvantage. However, the same opposing gender differences increase the gender gap in unhealthy life years (ULYs)^2^^,^^3^^,^^6^ and gender differences in ULY may even be larger than gender differences in life expectancy.^3^

Insight into the gender gap in ULY and the role of differences in mortality and disability, both in general and from specific chronic conditions, is relevant to better understand gender disparities and to optimize strategies to reduce these inequalities. A high contribution of a specific disease may point to specific risk factors, possibly modifiable.

The aim of this study is to improve understanding of gender disparities in ULY by quantifying the contribution of mortality and disability differences to the origin of gender gap in ULYs, both in total and from different causes of death and disability. We will address the following questions: (i) what is the contribution of women’s lower mortality (extending the time at risk of disability) and higher prevalence of disability to the gender gap in ULY and (ii) which causes of death and disability contribute most to this gender difference. We focus on France, given the availability of a large survey that comprises both the household and institutional population and includes a large set of conditions, including several mental disorders lacking in most previous studies.

## Methods

### Data

Table 1 presents the proportional mortality and disability for each disease group.

**Table 1:** Mortality and disability by disease group, age group and sex, France, 2008 based on CépiDc-INSERM-database^8^ and Disability Health Survey^9^

	Deaths by cause, % of total						Disability by cause, % of total					
	Men			Women			Men			Women		
	50–64	65–84	85+	50–64	65–84	85+	50–64	65–84	85+	50–64	65–84	85+
Heart disease	12.4	16.5	23.6	7.4	8.7	11.1	9.9	14.8	15.4	4.9	8.9	9.6
CVA	2.8	5.3	7.0	3.3	3.7	5.0	1.6	3.9	4.1	1.0	1.9	2.3
PVD	0.3	0.6	0.8	0.1	0.1	0.2	2.5	3.4	2.7	1.0	1.1	1.3
Other CVD	2.1	2.9	3.7	1.6	1.7	2.1	n.a.	n.a.	n.a.	n.a.	n.a.	n.a.
Musculoskeletal	0.3	0.5	0.7	0.6	0.6	0.8	28.3	21.1	15.5	38.9	33.5	19.9
Cancer	50.3	44.9	25.9	57.6	57.0	51.8	3.1	5.4	2.7	5.2	4.5	2.6
Alzheimer/Parkinson	0.4	3.3	8.3	0.7	1.3	2.7	1.0	2.3	7.3	1.0	3.0	7.5
Other neurological	2.0	1.6	0.9	3.0	2.9	2.6	2.4	1.3	2.2	2.1	1.6	0.9
CNSLD	1.3	2.3	3.0	0.9	1.1	1.4	6.4	6.1	6.3	4.4	3.7	2.4
Acute respiratory infec.	0.9	1.7	4.0	0.7	0.8	1.1	n.a.	n.a.	n.a.	n.a.	n.a.	n.a.
Anxiety and depression	0.2	0.1	0.3	0.3	0.3	0.3	6.5	3.6	3.2	10.8	8.2	4.7
Other mental diseases	3.1	0.8	0.3	1.8	1.5	0.9	1.7	0.9	0.8	0.7	0.4	0.7
Diabetes mellitus	1.6	2.5	2.3	1.6	2.0	2.6	3.1	3.2	1.9	4.0	4.8	2.8
Accidents	12.7	11.8	14.0	12.1	11.7	11.8	6.3	3.8	1.9	2.1	1.9	1.4
Other/background	9.5	5.2	5.3	8.4	6.6	5.3	27.4	29.9	36.1	23.9	26.3	43.7
Total	100	100	100	100	100	100	100	100	100	100	100	100

Note: CVA = cerebrovascular accident (cerebrovascular disease according to ICD-10 terminology). PVD = peripheral vascular disease. Other CVD = other cardiovascular diseases. CNSLD = chronic non-specific lung disease. Alzheimer’s/Parkinson groups include dementia. Anxiety and depression are mood disorders. n.a. = not distinguished as separate cause of disability.

Deaths and population by age and gender for the year 2008 for France from Eurostat were derived from the Eurohex website.^7^

Mortality and disability data by cause are presented in table 1. Mortality data by underlying cause of death were obtained from the CépiDc-INSERM-database.^8^

Data on disability by cause were derived in a previous paper^9^ based on the French Disability Health Survey (‘Enquête Handicap et Santé’) 2008–09 which consists of a survey among persons living in private households, HSM and a survey among persons living in in people living in nursing homes, homes for the elderly and mental institutions. Further details of the Disability Health Survey 2008–09 can be found elsewhere.^10^^,^^11^ Disability prevalence by cause was derived using the attribution method based on the additive hazard model.^1^^,^^12^ This method takes into account multi-morbidity and that people without reported diseases can still report disability. Further details on the method to estimate disability by cause are given in the Supplementary material.

### Definition of disability

To define disability we used the Global Activity Limitation Indicator (GALI), which is used to calculate HLY across Europe for health monitoring and target setting.^5^^,^^13^ The GALI is a single question ‘For at least the last 6 months, have you been limited because of a health problem in activities people usually do?’ aiming to capture long-term limitation (>6 months), and with three severity levels: none, limited but not severely and severely limited. The reliability and validity of the GALI have already been reported.^14–19^ People were considered to be disabled (unhealthy) when they reported any limitation. This is the cut-off used in the HLY measure in Europe.

### Causes of disability

We included the following groups of chronic conditions as causes of disability: heart diseases, cerebrovascular accident, peripheral vascular disease (PVD), cancer, chronic non-specific lung diseases (CNSLD), musculoskeletal diseases, Alzheimer’s and Parkinson’s disease, other neurological diseases (multiple sclerosis, and other unspecific neurological problems), depression and anxiety, other mental diseases (autism, schizophrenia and other unspecified psychiatric impairments), diabetes mellitus and accidents. These causes were selected considering the availability of diseases in the survey and main disability causes in prior research.^6^^,^^20^^,^^21^Supplementary table S1 presents the disease groups and diseases within each group.

### Cause of death

We grouped causes of death similarly to the standard groupings for causes of death, however because not all diseases that are disabling are fatal (and vice-versa) we made some adjustments. For completeness we included causes of death that do not cause long-term disability or were not distinguished in the disability survey, such as acute respiratory infections and other circulatory diseases. Causes of disability that are not common causes of death, such as musculoskeletal disorders, are included in the cause of death classification, but with virtually absent mortality.

### Life table and decomposition methods

Life expectancy with GALI disability, i.e. ULYs at age 50 for men and women were estimated by the Sullivan method. This method uses the gender-specific prevalence of disability in each age group to divide the number of person-years years in the standard life table into years with and without disability.^16^^,^^22^

The contribution of specific conditions to gender difference in ULYs was estimated by a life table decomposition tool which partitions the difference in ULYs into additive contributions of causes.^1^ The decomposition analysis assessed the difference in ULY because of smaller (higher) total mortality rates and/or disability prevalence (by age) from a given cause, in women relative to men. First, the difference in the number of unhealthy person-years (by age) was decomposed into two parts: the first part reflecting the smaller (larger) number of person-years (‘mortality effect’) and the second part reflecting the smaller (higher) prevalence of disability (‘disability effect’). Second, the mortality and disability effects were decomposed by age. For mortality, the decomposition method distinguishes between the age of origin (i.e. the age where mortality differences originate) and the age at destination (i.e. age groups to which person-years are added or removed). For decomposition by age, the age at origin is used,^1^ which gives the same result as the decomposition method of Andreev et al.^23^ Third, each age effect was decomposed by cause (disease contribution). The decomposition tool in R, including the decomposition by age, is provided in the Supplementary material. The decomposition analyses also output the decomposition of HLY, which is presented as Supplementary material (Supplementary table S2) for comparison.

## Results

Life expectancy of women aged 50 was 36.3 (95% CI 36.3–36.4) years and of men 30.4 years (95% CI 30.4–30.4) (table 2). Life expectancy with GALI disability was 19.0 years (95% CI: 18.4–19.6) for women compared to 14.2 years (95% CI 13.6–14.8) for men. The gender gap in total life expectancy was 5.9 years (95% CI 5.9–6.0) of which 4.8 (95% CI 4.0–5.7) were ULY.

**Table 2:** Gender difference in ULYs at age 50, and the contribution of mortality and disability differences for men and women, France, 2008

	ULY
	Years, (95% CI)
Men	14.2 (13.6 to 14.8)
Women	19.0 (18.4 to 19.6)
Difference	
Women−men	4.8 (4.0 to 5.7)
Contribution of mortality and disability differences	
Mortality effect	4.0 (3.9 to 4.2)
Disability effect	0.8 (−0.1 to 1.7)

Table 2 also shows the contribution of mortality differences (‘mortality effect’) and disability differences (‘disability effect’) to the gender difference in ULY. The lower mortality and higher disability effect increased ULY in women relative to men. The contribution of the mortality effect was 4.0 years and the disability effect was 0.8 year.

Further decomposition of the mortality effect and disability effect of ULY by cause is presented in table 3. Of the total mortality effect of 4.0 ULYs, 1.8 ULYs were due to women’s lower mortality from cancer, 0.8 ULY from heart disease and 0.3 ULY from accidents. Table 3 also shows gender differences in the disability effects of different diseases. Higher disability from musculoskeletal diseases and anxiety-depression for women than men increased the gender gap in ULY by 1.8 and 0.6 ULY, respectively. The gender gap in ULY was reduced due to lower disability in women from: heart disease by 0.8 ULY, CNSLD by 0.4 ULY and accidents by 0.3 ULY. The sum of the mortality and disability effects showed that musculoskeletal diseases (due to their higher contribution of disability in women) and cancer (due to their lower contribution of mortality in women) had the greatest contribution to the longer ULY in women.

**Table 3:** Decomposition of gender differences (women−men) in life expectancy with disability (ULY) at age 50, into mortality and disability effects and total effect by cause, France 2008

	Mortality effect (95% CI)	Disability effect (95% CI)	Total effect (95% CI)
Cardiovascular diseases			
Heart disease	0.76 (0.75 to 0.77)	−0.82 (−1.37 to −0.27)	−0.06 (−0.60 to 0.49)
CVA	0.13 (0.13 to 0.14)	−0.23 (−0.45 to 0.00)	−0.10 (−0.31 to 0.13)
PVD	0.03 (0.03 to 0.03)	−0.26 (−0.48 to −0.07)	−0.23 (−0.44 to −0.03)
Other cardiovascular diseases	0.10 (0.10 to 0.10)	n.a	0.10 (0.10 to 0.10)
Musculoskeletal diseases	0.00 (0.00 to 0.00)	1.84 (0.96 to 2.94)	1.84 (0.96 to 2.94)
Cancer	1.75 (1.74 to 1.77)	0.09 (−0.23 to 0.43)	1.84 (1.54 to 2.18)
Neurological diseases			
Alzheimer/Parkinson	0.04 (0.04 to 0.04)	0.08 (−0.17 to 0.28)	0.12 (−0.14 to 0.32)
Other neurological diseases	0.05 (0.04 to 0.05)	−0.04 (−0.22 to 0.12)	0.00 (−0.17 to 0.17)
Respiratory diseases			
CNSLD	0.14 (0.14 to 0.14)	−0.41 (−0.84 to 0.03)	−0.27 (−0.70 to 0.17)
Acute respiratory infections	0.10 (0.09 to 0.10)	n.a	0.10 (0.09 to 0.10)
Mental diseases			
Anxiety and depression	0.00 (0.00 to 0.00)	0.63 (0.20 to 1.04)	0.63 (0.20 to 1.04)
Other mental diseases	0.06 (0.06 to 0.07)	−0.07 (−0.22 to 0.03)	−0.01 (−0.01 to −0.02)
Diabetes mellitus	0.08 (0.07 to 0.08)	0.22 (−0.15 to 0.59)	0.30 (−0.07 to 0.66)
Accidents	0.28 (0.27 to 0.28)	−0.32 (−0.63 to −0.02)	−0.05 (−0.35 to 0.26)
Other/background	0.50 (0.48 to 0.51)	0.08 (−0.27 to 0.33)	0.58 (0.22 to 0.83)
Total	4.0 (3.9 to 4.2)	0.8 (−0.1 to 1.7)	4.8 (4.0 to 5.7)

Note: CVA = cerebrovascular accident (corresponding to cerebrovascular diseases according to the ICD-10 terminology). PVD = periphery vascular diseases. CNSLD = chronic non-specific lung disease. n.a = not available, no separate cause of long-term disability. Gender differences in ULY can originate from higher disability and/or from lower mortality from the condition (extending the time at risk of disability from any cause). The cause-specific mortality effects indicate the origin of the female excess in overall ULY resulting from lower female mortality, although these diseases are not the causes of the ULY themselves. The total effect refers to the disease-specific origin of the gender differences in ULY, either by higher (lower) disability from this condition, of by lower mortality from this condition, extending the time at risk of disability from any conditions.

## Discussion

### Summary of findings

In our study, French women spend 4.8 more years with disability than men, mainly because women live longer and are exposed to disability in these additional years. This survival advantage in women reflects their lower mortality from cancer and cardiovascular diseases, and to a smaller extent accidents and respiratory diseases, which together explained nearly 75% of women’s excess ULY. In addition, a small disability effect, reflecting higher prevalence of disability in women, contributed to <1 excess ULY in women. This disability effect results from the opposing contributions of specific conditions: musculoskeletal diseases and anxiety-depression contributed more to disability in women than men; and cardiovascular diseases, CNSLD and accidents contributed more to disability in men.

### Strengths and weaknesses

The strength of our study includes considering disparities in both disability and mortality and using a very comprehensive and large survey on disability and disease in the entire population. This study used disability data by cause derived from a survey of people living in private households and institutions and included a wide range of conditions including the consequences of injuries and mental diseases. We also had access to very detailed cause-of-death data, which allowed us to make cause of death groups that best matched with the diseases groups in the survey.

Some limitations of our study must be considered. First, data on disability and diseases were self-reported. However, for most chronic diseases, self-report is fairly accurate,^24^^,^^25^ with lowest accuracy for arthritis.^24^^,^^26^ Moreover, there is no consistent evidence that men and women form assessments of their health in different ways.^27^ Higher disability prevalence in women was also found in several performance-based measures.^28^^,^^29^ Second, in contrast to the causes of death, which are obtained from the death certificate, the causes of disability in our study were based on a statistical attribution of disability to diseases based on cross-sectional survey data. While this attribution took into account competing causes of disability and the presence of disability in people without any diseases, we cannot ascertain that all diseases were present before the onset of disability. In particular for depression, it cannot be ruled out that disability is the cause and not the result of depression.^30^ Third, our data did not allow us to use a cohort perspective nor to study changes over time. The Sullivan method uses a period perspective which involves the stationary assumptions,^31^ i.e. constant age-specific hazard rates and constant births equal to the radix of the life table. Simulation studies have shown that these assumptions have a minor influence on the results, unless large changes have occurred in mortality and/or disability in the study period.^31–34^ Therefore, the Sullivan method is routinely used to calculate life expectancy with(out) disability. For the decomposition method, based on the Sullivan method, it is noteworthy that it identifies the extent to which differences in disability prevalence and total mortality (in each age group) contribute to ULY differences, but not the contribution of differences in incidence of disability, recovery from disability and survival.^1^

### Interpretation and comparison with prior studies

The decomposition analyses assessed which conditions contributed to excess ULY in women. The focus was the origin of these differences and we took into account the excess ULY that can originate from higher disability and/or from lower mortality from the condition (extending the time at risk to disability from any cause). The latter does not provide insight into the conditions that contribute to the ULY resulting from the lower female mortality.

Women experience lower mortality, even after the advent of ill-health,^35^ and explanations for this include biological (hormonal and genetic), behavioral and social differences.^25^^,^^29^^,^^36^ It is plausible that the same factors explain both lower mortality and lower disability from cardiovascular diseases (e.g. hormonal, genetic, smoking, alcohol consumption and diet), respiratory diseases (e.g. smoking) and accidents (e.g. risk taking and alcohol consumption), supported by the finding that the lower contribution of these conditions to disability in women was due to lower disease prevalence since the disabling impacts of these conditions did not differ by gender.^9^ Our study found opposite contributions for musculoskeletal diseases, diabetes and mental diseases. Compared to men, higher disability in women from these conditions contributed to more ULY in women. For musculoskeletal diseases and diabetes this reflected both higher disease prevalence and higher disabling impact in women than in men,^9^ perhaps due to higher frequency of obesity and lower levels of physical activity in French women.^37^ Differences in body composition (bone mass, muscle strength) and hormonal differences may also have contributed to a higher susceptibility of women to musculoskeletal diseases.^38^ The higher contribution of mental diseases to disability in women than men mainly reflected differences in disease prevalence.^9^ These gender differences in the contribution of mental disease are widely acknowledged but less understood.^39^

Similar to our study, prior decomposition analyses of gender differences in life expectancy with disability^1–3^^,^^6^ showed a larger gender gap in ULY than in HLY due to the combination of lower mortality and higher disability, both extending ULY in women as compared to men. Two previous studies looked at the contributions of diseases to gender differences in life expectancy with disability.^1^^,^^6^ Our study confirmed that arthritis contributed most to the longer life expectancy with disability in women than in men because of the disability effect while cancer was the main contributor to longer ULY in women because of the mortality effect. However, the earlier Dutch study^1^ showed no contribution from heart diseases to the disability effect, while the Belgium study^6^ confirmed our findings of a lower contribution of heart diseases to disability in women for the year 2008, and additionally showed that this effect changed over time from a higher contribution in 2001 for women than for men, to a lower contribution in more recent periods. The differences with the Dutch findings may also reflect differences in timing of the study and the study population. The Dutch study^1^ used data from the early 1990s, and since then survival from heart disease has increased substantially, persons who previously died from the disease are now more likely to survive. In addition, the Dutch study^1^ excluded the institutionalized population which may explain the absence of the gender difference in the contribution of heart disease.^9^

Prior research on the male–female health-survival paradox has pointed to the role of different diseases: men are more likely to die from fatal diseases and women suffer more from non-fatal diseases.^4^^,^^25^^,^^27^^,^^40^ Our study confirmed that higher disability from non-fatal diseases, such as musculoskeletal diseases and anxiety-depression in women contributed to more ULYs in women, but that this effect was partly nullified by higher prevalence of disability in men from the traditional life-threatening diseases, such as cardiovascular diseases, respiratory diseases and accidents. A second explanation for the health-survival paradox given in the literature is that women live longer and for that reason face more disability.^4^ Our study confirmed that lower mortality tended to increase ULY in women relative to men, and added that this mortality effect was larger than the disability effect.

### Conclusions, implications and recommendations

We find that both elements of the health-survival paradox, higher disability and lower mortality, contribute to the excess ULYs in French women compared to men, although women’s mortality advantage was the main contributor. However, within the small net contribution of the disability effect, diseases such as musculoskeletal diseases or anxiety-depression have a substantial positive contribution, though this is offset by the negative contribution of heart diseases, respiratory diseases and accidents. Diseases that are traditionally seen as male fatal diseases also cause disability in men and tend to reduce the gender gap in ULY.

Our study shows that diseases have a different impact on gender differences in ULY, depending on whether the disease is mainly life-threatening (such as cancer), mainly disabling (such as musculoskeletal) or both (such as heart diseases). A life-threatening disease reduces ULY as people live shorter lives and therefore are less exposed to disability from any cause. A disabling disease increases ULY as persons experience disability from this disease. Whether a disease which is both life-threatening and disabling reduces or increases ULY depends on the relative size of both effects.

An implication of our findings for the strategy to reduce the gender gap in ULY is that the largest reduction in the female disadvantage in ULY could be obtained by reducing male mortality disadvantage. However, this would not reduce ULY in women and would increase ULY in men in the total population. Instead, there is a strong need to target prevention of diseases that cause disability, including musculoskeletal diseases which contribute most to disability in both men and women. This includes policies and interventions that reduce obesity by targeting sedentary behavior, physical inactivity and unhealthy diets. Moreover, reducing the disabling consequences of diseases is increasingly important, as people live longer with diseases that cause disability. Better disease management, more use of assistive devices and technological developments and adaptation of the environment are examples to reduce the burden of disability in people with diseases. This may not only reduce the gender gap in life expectancy with disability, but at the same time avoid future increases in disability for both genders.

## Funding

This work was funded by the Caisse Nationale de Solidarité de Autonomie (CNSA). This research was also part of the European Joint-Action on European Health and Life Expectancy Information System (JA-EHLEIS), funded by the Executive Agency for Health and Consumer of the European Commission (agreement number 2010 23 01). The research finally benefitted from cross collaborations with the research program ISHEF (IRESP) [n° AAP-2011-01].

*Conflicts of interest*: None declared.

**Key points**

- French women’s disadvantage in ULYs compared to men is predominantly due to women’s lower mortality from cancer, cardiovascular and respiratory diseases and accidents.
- Women’s higher disability contributes moderately to their excess ULYs.
- The substantial disabling effect of musculoskeletal diseases in women’s ULY is offset by the lower contribution of heart and respiratory diseases, and accidents compared to men.

## Supplementary Material

ckz114_Supplementary_Data

## References

[1] Nusselder WJ, Looman CW Decomposition of differences in health expectancy by cause. Demography 2004;41:315–34. 15209043 10.1353/dem.2004.0017

[2] Nusselder WJ, Looman CW, Van Oyen H, et al Gender differences in health of EU10 and EU15 populations: the double burden of EU10 men. Eur J Ageing 2010;7:219–27. 21212821 10.1007/s10433-010-0169-x PMC2995874

[3] Van Oyen H, Nusselder W, Jagger C, et al Gender differences in healthy life years within the EU: an exploration of the “health-survival” paradox. Int J Public Health 2013;58:143–55. 22618297 10.1007/s00038-012-0361-1 PMC3557379

[4] Luy M, Minagawa Y Gender gaps - Life expectancy and proportion of life in poor health. Health Rep 2014;25:12–19. 25517936

[5] Jagger C, McKee M, Christensen K, et al Mind the gap–reaching the European target of a 2-year increase in healthy life years in the next decade. Eur J Public Health 2013;23:829–33. 23487547 10.1093/eurpub/ckt030 PMC3784798

[6] Yokota RTC, Nusselder WJ, Robine JM, et al Contribution of chronic conditions to gender disparities in health expectancies in Belgium, 2001, 2004 and 2008. Eur J Public Health 2018;29:82–7. 10.1093/eurpub/cky105 29917065

[7] Eurohex. Advanced Research on Health Expectancies. Eurohex, 2016 Available at: http://www.eurohex.eu/ (18 December 2017, date last accessed).

[8] Mesle F CépiDc-INSERM Database. CépiDc-INSERM, 2008 Available at: https://www.cepidc.inserm.fr/.

[9] Nusselder WJ, Wapperom D, Looman CWN, et al Contribution of chronic conditions to disability in men and women in France. Eur J Public Health 2018;29:99–104. 10.1093/eurpub/cky138 30107556

[10] INSEE. Enquête Handicap-santé - Volet Institutions 2009/HSI. INSEE, 2016. Available at: https://www.insee.fr/fr/metadonnees/source/s1244 (28 April 2016, date last accessed).

[11] INSEE. Enquête Handicap-santé 2008 - Volet Ménages/HSM. INSEE, 2016. Available at: https://www.insee.fr/fr/metadonnees/source/s1245 (25 March 2016, date last accessed).

[12] Yokota RTC, Van Oyen H, Looman CWN, et al Multinomial additive hazard model to assess the disability burden using cross-sectional data. Biom J 2017;59:901–17. 28332222 10.1002/bimj.201600157

[13] Lagiewka K European innovation partnership on active and healthy ageing: triggers of setting the headline target of 2 additional healthy life years at birth at EU average by 2020. Arch Public Health 2012;70:23 23088612 10.1186/0778-7367-70-23 PMC3492155

[14] Cox B, van Oyen H, Cambois E, et al The reliability of the Minimum European Health Module. Int J Public Health 2009;54:55–60. 19183846 10.1007/s00038-009-7104-y

[15] Van Oyen H, Van der Heyden J, Perenboom R, Jagger C Monitoring population disability: evaluation of a new Global Activity Limitation Indicator (GALI). Soz Praventivmed 2006;51:153–61. 17191540 10.1007/s00038-006-0035-y

[16] Jagger C, Gillies C, Cambois E, et al The Global Activity Limitation Index measured function and disability similarly across European countries. J Clin Epidemiol 2010;63:892–9. 20171842 10.1016/j.jclinepi.2009.11.002

[17] Van der Heyden J, Berger N, Van Oyen H Comparison of self-rated health and activity limitation as predictors of short term mortality in the older population. Public Health 2015;129:283–5. 25694238 10.1016/j.puhe.2014.12.005

[18] Berger N, Van Oyen H, Cambois E, et al Assessing the validity of the Global Activity Limitation Indicator in fourteen European countries. BMC Med Res Methodol 2015;15:1. 25555466 10.1186/1471-2288-15-1 PMC4298058

[19] Van Oyen H, Bogaert P, Yokota RTC, Berger N Measuring disability: a systematic review of the validity and reliability of the Global Activity Limitations Indicator (GALI). Arch Public Health 2018;76:25. 29881544 10.1186/s13690-018-0270-8 PMC5985596

[20] Nusselder WJ, Looman CW, Mackenbach JP, et al The contribution of specific diseases to educational disparities in disability-free life expectancy. Am J Public Health 2005;95:2035–41. 16195519 10.2105/AJPH.2004.054700 PMC1449480

[21] Klijs B, Nusselder WJ, Looman CW, Mackenbach JP Contribution of chronic disease to the burden of disability. PLoS One 2011;6:e25325. 21966497 10.1371/journal.pone.0025325 PMC3178640

[22] Jagger C, Van Oyen H, Robine JM Health Expectancy Calculations by the Sullivan Method: a Practical Guide. 2014.

[23] Andreev E, Shkolnikov V, Begun A Algorithm for decomposition of differences between aggregate demographic measures and its application to life expectancies, healthy life expectancies, parity progression ratios and total fertility rates. Demogr Res 2002;7:499–522.

[24] Oksanen T, Kivimaki M, Pentti J, et al Self-report as an indicator of incident disease. Ann Epidemiol 2010;20:547–54. 20538198 10.1016/j.annepidem.2010.03.017

[25] Crimmins EM, Kim JK, Sole-Auro A Gender differences in health: results from SHARE, ELSA and HRS. Eur J Public Health 2011;21:81–91. 20237171 10.1093/eurpub/ckq022 PMC3023013

[26] Kriegsman DM, Penninx BW, van Eijk JT, et al Self-reports and general practitioner information on the presence of chronic diseases in community dwelling elderly. A study on the accuracy of patients' self-reports and on determinants of inaccuracy. J Clin Epidemiol 1996;49:1407–17. 8970491 10.1016/s0895-4356(96)00274-0

[27] Case A, Paxson C Sex differences in morbidity and mortality. Demography 2005;42:189–214. 15986983 10.1353/dem.2005.0011

[28] Merrill SS, Seeman TE, Kasl SV, Berkman LF Gender differences in the comparison of self-reported disability and performance measures. J Gerontol A Biol Sci Med Sci 1997;52:M19–26. 9008665 10.1093/gerona/52a.1.m19

[29] Oksuzyan A, Guma J Sex differences in health and survival In: Guma J, editor. A Demographic Perspective on Gender, Family and Health in Europe. Cham, Switzerland: Springer, 2018: 65–100.

[30] Whiteford HA, Degenhardt L, Rehm J, et al Global burden of disease attributable to mental and substance use disorders: findings from the Global Burden of Disease Study 2010. Lancet 2013;382:1575–86. 23993280 10.1016/S0140-6736(13)61611-6

[31] Imai K, Soneji S On the estimation of disability-free life expectancy: Sullivan' method and its extension. J Am Stat Assoc 2007;102:1199–211. 26279593 10.1198/016214507000000040 PMC4533834

[32] Barendregt JJ, Bonneux L, Van der Maas PJ Health expectancy: an indicator for change? Technology Assessment Methods Project Team. J Epidemiol Community Health 1994;48:482–7. 7964359 10.1136/jech.48.5.482 PMC1060012

[33] Van de Water HP, Boshuizen HC, Perenboom RJ, et al Health expectancy: an indicator for change? J Epidemiol Community Health 1995;49:330–1. 7629477 10.1136/jech.49.3.330 PMC1060811

[34] Mathers CD, Robine JM How good is Sullivan's method for monitoring changes in population health expectancies? J Epidemiol Community Health 1997;51:80–6. 9135793 10.1136/jech.51.1.80 PMC1060414

[35] Hohn A, Larsen LA, Schneider DC, et al Sex differences in the 1-year risk of dying following all-cause and cause-specific hospital admission after age 50 in comparison with a general and non-hospitalised population: a register-based cohort study of the Danish population. BMJ Open 2018;8:e021813 10.1136/bmjopen-2018-021813 PMC6059308 30018099

[36] Rogers RG, Everett BG, Onge JM, Krueger PM Social, behavioral, and biological factors, and sex differences in mortality. Demography 2010;47:555–78. 20879677 10.1353/dem.0.0119 PMC3000060

[37] DREES. La santé des femmes. 2013 Available at: https://drees.solidarites-sante.gouv.fr/IMG/pdf/er834.pdf.

[38] Woolf AD, B P Burden of major musculoskeletal conditions. Bull World Health Organ 2003;81:646–56. 14710506 PMC2572542

[39] Riecher-Rossler A Sex and gender differences in mental disorders. Lancet Psychiatry 2017;4:8–9. 27856397 10.1016/S2215-0366(16)30348-0

[40] Oksuzyan A, Juel K, Vaupel JW, Christensen K Men: good health and high mortality. Sex differences in health and aging. Aging Clin Exp Res 2008; 20:91–102. 18431075 10.1007/bf03324754 PMC3629373

