# Supplementary material for: Women’s excess unhealthy life years: disentangling the unhealthy life years gap
Source: Eur J Public Health. 2019 Jul 5;29(5):914–9. doi: 10.1093/eurpub/ckz114 (PMC6761840; doi:10.1093/eurpub/ckz114)
Supplement: ckz114_Supplementary_Data [file ckz114_supplementary_data.zip › ckz114-Suppl_data/Supplementary_Data1.docx]

Supplementary Table S1 Chronic disease groups and the included diseases as causes of death and causes of disability

| **Groups** | **Causes of disability** | **Causes of mortality (ICD 10)** | **Codes CépiDc** |
| --- | --- | --- | --- |
| Cancer | Cancer | C00-C26, C30-C31, C32-C34, C37-C49; C50; C51-C60; C61; C62-D48 | 1510; 1610; 1710; 1810; 1910; 2010 |
| Heart diseases | Heart diseases | I00-I09; I20-I25; I26-I46; I47-I49; 9; I50; I51; | 0101;0202; 505; 1108; 1300; |
| Cerebrovascular accidents | Cerebrovascular accident | I60-I69 | 0404 |
| Periphery vascular disease | Periphery vascular disease | I70; I776 | 0606;0806 |
| Other cardiovascular diseases | - | I10-I15; I71-I72; I73-I775; I777-I78; I80-I82;I83-I84; I85-I99 | 0303; 0706; 0906; 1007;1209; 1400 |
| CNSLD | CNSLD | J40-J47 | 2111;2112 |
| Acute respiratory (Influenza/ pneumonia/ acute bronchitis) | - | J00-J22 | 2300 |
| Diabetes Mellitus | Diabetes Mellitus | E10-E14 | 2626 |
| Alzheimer-Parkinson-Dementia* | Alzheimer/Parkinson/  Dementia | F1-F3; G20-G21; G30-G31 | 3130;3231 |
| Other neurological | Other neurological: multiple sclerosis, other unspecific neurological problems | G00-G12; G23-G25; G35; G36-G37; G40-G41; G43; G45-G98 | 3029; 3449; 3332 |
| Mental diseases** | Anxiety-depression | F30-F48 | 3500 |
|  | Other mental diseases: autism, schizophrenia, other unspecified psychiatric impairments | F04-F29 | 3600 |
| Musculoskeletal | Musculoskeletal | M00-M99 | 2500 |
| Accidents/Injury*** | Consequences of injury | V01-Y89 | 3748 |
| Other | Other: all other diseases on disease card | A00-B99; D50-D98; E00-E07; H00-H95; G43-G44; K00-K93; L00-L98; N00-N99; O00-O99; P00-P96; Q00-Q99; R00-R99 | 4600; 4700;2400; 2727;2800; 2928; 3823; 3924; 4025;4100; 4200; 4300; 4400; 4500; 4800; 4900 |

******* Alzheimer’s/Parkinson groups includes dementia

**Anxiety-depression are mood disorders.

***Accidents include all external causes of death

**Supplementary material: Decomposition tool**

The R program with the decomposition tool is available in a separate file:

Decomposition tool Nusselder v_Jan2019.r

The User manual is available at: <http://www.eurohex.eu/pdf/Decompostion%20guide.pdf>

Example input files can be obtained from the author: w.nusseldder@erasmusmc.nl

**Supplementary material: Attribution method**

The attribution method (1, 2), based on the additive hazard model, was used. This method takes into account that people who do not report a disease may be disabled (this is referred to as “background”) and that people may have more than one disease (multi-morbidity). Disability in people without a reported disease is entirely attributed to background. Disability in people with at least one disease is attributed partly to background and partly to the disease(s). We assumed that: 1) the distribution of disability by cause is explained entirely by diseases that are (still) present at the time of the survey, 2) that these diseases and background act as independent competing causes, 3) the distribution across different causes is proportionally equal to the cumulative rates of becoming disabled from each cause and 4) the start of the time at risk for disability is the same for all diseases (in the same age group and gender).

The regression model is specified as follows:

where is the estimated probability that the person has disability and the linear predictor. The latter is defined as the sum of the background cumulative rate by 5-yeear age () and the disease-specific cumulative rates of disability (, labeled as “disabling impact”) for the disease groups () that are present in the respondent (given by the dummy variables ) by 15 year age groups. Background was handled as a cause that is present for everyone, irrespective of the presence of disease(s). Disability attributed to disease is and to background is (1).

Applying these formulas gives for every individual the probability of being disabled caused by background *or* disease (if present). Adding the cause-specific probabilities of an individual gives the probability of being disabled for that individual.

For the attribution of diseases to disability, we allowed the disabling impacts to vary by age (). As the full age-disease interaction term would require (number of age classes) times (number of diseases) different parameters, the rank of the interaction between age and diseases was reduced using Reduced Rank Regression (3).

The attribution methods was applied in prior publications (1, 2, 4-12).

1. Nusselder WJ, Looman CW. Decomposition of differences in health expectancy by cause. Demography. 2004;41(2):315-34.

2. Yokota RTC, Van Oyen H, Looman CWN, Nusselder WJ, Otava M, Kifle YW, et al. Multinomial additive hazard model to assess the disability burden using cross-sectional data. Biom J. 2017;59(5):901-17.

3. Yee T, Hastle T. Reduced-rank vector generalized linear models. Statistical modelling. 2003;3(1):15-41.

4. Yokota RT, Berger N, Nusselder WJ, Robine JM, Tafforeau J, Deboosere P, et al. Contribution of chronic diseases to the disability burden in a population 15 years and older, Belgium, 1997-2008. BMC Public Health. 2015;15:229.

5. Yokota RT, de Moura L, Andrade SS, de Sa NN, Nusselder WJ, Van Oyen H. Contribution of chronic conditions to gender disparities in disability in the older population in Brazil, 2013. International journal of public health. 2016;61(9):1003-12.

6. Yokota RT, Nusselder WJ, Robine JM, Tafforeau J, Deboosere P, Van Oyen H. Contribution of Chronic Conditions to the Disability Burden across Smoking Categories in Middle-Aged Adults, Belgium. PLoS One. 2016;11(4):e0153726.

7. Yokota RT, Van der Heyden J, Demarest S, Tafforeau J, Nusselder WJ, Deboosere P, et al. Contribution of chronic diseases to the mild and severe disability burden in Belgium. Arch Public Health. 2015;73(1):37.

8. Yokota RT, Van der Heyden J, Nusselder WJ, Robine JM, Tafforeau J, Deboosere P, et al. Impact of Chronic Conditions and Multimorbidity on the Disability Burden in the Older Population in Belgium. J Gerontol A Biol Sci Med Sci. 2016;71(7):903-9.

9. Klijs B, Nusselder WJ, Looman CW, Mackenbach JP. Contribution of chronic disease to the burden of disability. PLoS One. 2011;6(9):e25325.

10. Nusselder WJ, Looman CW, Mackenbach JP, Huisman M, van Oyen H, Deboosere P, et al. The contribution of specific diseases to educational disparities in disability-free life expectancy. Am J Public Health. 2005;95(11):2035-41.

11. Klijs B, Nusselder WJ, Looman CW, Mackenbach JP. Educational disparities in the burden of disability: contributions of disease prevalence and disabling impact. Am J Public Health. 2014;104(8):e141-8.

12. Nusselder WJ, Wapperom D, Looman CWN, Yokota RTC, van Oyen H, Jagger C, et al. Contribution of chronic conditions to disability in men and women in France. European journal of public health. 2018.

Table S2 Decomposition of gender differences in total life expectancy, disability-free life expectancy (HLY), and life expectancy with disability (ULY) at age 50, into mortality and disability effects and total effect by cause, France 2008

|  | LE |  | HLY |  |  | | ULY |  |
| --- | --- | --- | --- | --- | --- | --- | --- | --- |
|  | Mortality effect | Mortality effect | Disability effect | Total effect | Mortality effect | Disability effect | | Total effect |
| Circulatory r diseases | | |  |  |  |  | |  |
| Heart disease | 1.11 CI(1.09; 1.12) | 0.34 CI(0.32; 0.37) | 0.82 CI(0.27; 1.36) | 1.17 CI(0.48; 1.58) | 0.76 CI(0.75; 0.77) | -0.82 CI(-1.37; -0.27) | | -0.06 CI(-0.60; 0.49) |
| Other circulatory diseases | 0.15 CI(0.15; 0.15) | 0.05 CI(0.05; 0.05) | | 0.05 CI(0.05; 0.05) | 0.10 CI(0.10; 0.10) | | | 0.10 CI(0.10; 0.10) |
| Cerebrovascular diseases | 0.20 CI(0.19; 0.20) | 0.06 CI(0.06; 0.07) | 0.23 CI(0.00; 0.45) | 0.29 CI(0.06; 0.51) | 0.13 CI(0.13; 0.14) | -0.23 CI(-0.45; 0.00) | | -0.10 CI(-0.31; 0.13) |
| Peripheral vascular disease | 0.05 CI(0.05; 0.05) | 0.01 CI(0.01; 0.01) | 0.26 CI(0.07; 0.48) | 0.27 CI(0.08; 0.49) | 0.03 CI(0.03; 0.03) | -0.26 CI(-0.48; -0.07) | | -0.23 CI(-0.44; -0.03) |
| Musculoskeletal diseases | 0.00 CI(0.00; 0.00) | 0.00 CI(0.00; 0.00) | -1.84 CI(-2.94; -0.96) | -1.84 CI(-2.94; -0.96) | 0.00 CI(0.00; 0.00) | 1.84 CI(0.96; 2.94) | | 1.84 CI(0.96; 2.94) |
| Cancer | 2.61 CI(2.58; 2.63) | 0.85 CI(0.80; 0.91) | -0.09 CI(-0.43; 0.23) | 0.77 CI(0.42; 1.08) | 1.75 CI(1.74; 1.77) | 0.09 CI(-0.23; 0.43) | | 1.84 CI(1.54; 2.18) |
| Neurological diseases | |  |  |  |  |  | |  |
| Alzheimer/ Parkinson | 0.05 CI(0.05; 0.06) | 0.02 CI(0.01; 0.02) | -0.08 CI(-0.28; 0.17) | -0.06 CI(-0.27; 0.19) | 0.04 CI(0.04; 0.04) | 0.08 CI(-0.17; 0.28) | | 0.12 CI(-0.14; 0.32) |
| Other neurological diseases | 0.07 CI(0.07; 0.07) | 0.02 CI(0.02; 0.03) | 0.04 CI(-0.12; 0.22) | 0.07 CI(-0.10; 0.24) | 0.05 CI(0.04; 0.05) | -0.04 CI(-0.22; 0.12) | | 0.00 CI(-0.17; 0.17) |
| Respiratory diseases | |  |  |  |  |  | |  |
| CNSLD | 0.19 CI(0.19; 0.19) | 0.05 CI(0.05; 0.06) | 0.41 CI(-0.03; 0.84) | 0.46 CI(0.02; 0.89) | 0.14 CI(0.14; 0.14) | -0.41 CI(-0.84; 0.03) | | -0.27 CI(-0.70; 0.17) |
| Acute respiratory infection | 0.13 CI(0.13; 0.13) | 0.03 CI(0.03; 0.04) | | 0.03 CI(0.03; 0.04) | 0.10 CI(0.09; 0.10) | | | 0.10 CI(0.09; 0.10) |
| Mental diseases | |  |  |  |  |  | |  |
| Anxiety and depression | 0.00 CI(0.00; 0.00) | 0.00 CI(0.00; 0.00) | -0.63 CI(-1.04; -0.20) | -0.63 CI(-1.04; -0.20) | 0.00 CI(0.00; 0.00) | 0.63 CI(0.20; 1.04) | | 0.63 CI(0.20; 1.04) |
| Other mental diseases | 0.11 CI(0.11; 0.11) | 0.05 CI(0.04; 0.05) | 0.07 CI(-0.03; 0.22) | 0.12 CI(0.02; 0.26) | 0.06 CI(0.06; 0.07) | -0.07 CI(-0.22; 0.03) | | -0.01 CI(-0.01; -0.02) |
| Diabetes Mellitus | 0.11 CI(0.11; 0.11) | 0.04 CI(0.03; 0.04) | -0.22 CI(-0.58; 0.15) | -0.19 CI(-0.55; 0.18) | 0.08 CI(0.07; 0.08) | 0.22 CI(-0.15; 0.59) | | 0.30 CI(-0.07; 0.66) |
| Accidents | 0.43 CI(0.43; 0.43) | 0.15 CI(0.15; 0.16) | 0.32 CI(0.02; 0.63) | 0.48 CI(0.17; 0.78) | 0.28 CI(0.27; 0.28) | -0.32 CI(-0.63; -0.02) | | -0.05 CI(-0.35; 0.26) |
| Other /background | 0.73 CI(0.73; 0.74) | 0.24 CI(0.22; 0.25) | -0.08 CI(-0.33; 0.27) | 0.16 CI(-0.10; 0.52) | 0.50 CI(0.48; 0.51) | 0.08 CI(-0.27; 0.33) | | 0.58 CI(0.22; 0.83) |
|  |  |  |  |  |  |  | |  |
| Total | 5.9 (5.9; 6.0) | 1.9 (1.8; 2.1) | -0.8 (-1.7; 0.1) | 1.1 (0.2; 2.0) | 4.0 (3.9; 4.2) | 0.8 (-0.1; 1.7) | | 4.8 (4.0; 5.7) |

CNSLD = chronic nonspecific lung disease

Mortality effect HLY = mortality effect LE – mortality effect ULY

Disability effect ULY is – (disability effect HLY)

Note: Gender differences in ULY can originate from higher disability and/or from lower mortality from the condition (extending the time at risk of disability from any cause). The cause-specific mortality effects indicate the origin of the female excess in overall ULY resulting from lower female mortality, although these diseases are not the causes of the ULY themselves. The total effect refers to the disease-specific origin of the gender differences in ULY, either by higher (lower) disability from this condition, of by lower mortality from this condition, extending the time at risk of disability from any conditions.
